# How effective are population health surveys for estimating prevalence of chronic conditions compared to anonymised clinical data?

**DOI:** 10.23889/ijpds.v5i1.1151

**Published:** 2020-06-12

**Authors:** T Whiffen, A Akbari, T Paget, S Lowe, R Lyons

**Affiliations:** Welsh Government; Health Data Research UK, Swansea University; Administrative Data Research Wales; Swansea University

## Abstract

**Introduction:**

Population health surveys are used to record person-reported outcome measures for chronic health conditions and provide a useful source of data when evaluating potential disease burdens. The reliability of survey-based prevalence estimates for chronic diseases is unclear nonetheless. This study applied methodological triangulation via a data linkage method to validate prevalence of selected chronic conditions (angina, myocardial infarction, heart failure, and asthma).

**Methods:**

Linked healthcare records were used for a combined cohort of 11,323 adults from the 2013 and 2014 sweeps of the Welsh Health Survey (WHS). The approach utilised consented survey data linked to primary and secondary care electronic health record (EHR) data back to 2002 within the Secure Anonymised Information Linkage (SAIL) Databank.

**Results:**

This descriptive study demonstrates validation of survey and clinical data using data linkage for selected chronic cardiovascular conditions and asthma with varied success. The results indicate that identifying cases for separate cardiovascular conditions was limited without specific medication codes for each condition, but more straightforward for asthma, where there was an extensive list of medications available. For asthma there was better agreement between prevalence estimates based on survey and clinical data as a result.

**Conclusion:**

Whilst the results provide external validity for the WHS as an instrument for estimating the burden of chronic disease, they also indicate that a data linkage appproach can be used to produce comparable prevalence estimates using clinical data if a defined condition-specific set of clinical codes are available.

## Introduction

Population health surveys have been used to record information on chronic conditions and self-reported health for many years [1]. For well-developed healthcare systems, patient reported health surveys provide a useful source of information for health research when this information is not collected within routine administrative data. Measures of chronic conditions from health surveys provide an indication of disease burden and potential need for health services for various conditions including those which carry a greater risk of mortality, for instance cardiovascular disease [2].

For many years, the Welsh Health Survey (WHS) was the main instrument for point-in-time measures of self-reported health and morbidity in Wales. In 2015 the WHS was incorporated into the revamped National Survey for Wales (NSW), but it could be argued that alternative methods of estimating chronic disease prevalence now exist. The warehousing of fairly comprehensive clinical datasets (derived from electronic health records (EHR)) from primary and secondary care in a secure research environment as provided by the Secure Anonymised Information Linkage (SAIL) Databank means that prevalence of diseases could potentially be measured on a whole population basis. The utility of the SAIL Databank has been expanded by storage of both healthcare and non-clinical data, such as survey data from the WHS and the NSW, in the same environment and with the potential to link these datasets together. Following these developments, it is now possible to address these research questions:

1. How do counts of chronic disease derived from clinical EHR compare with self-reported counts from the Welsh Health Survey?
2. Where a disease register does not exist, is it feasible to use an algorithm to identify prevalence of specific chronic conditions from EHR?

This study seeks to address these questions. Although not completely comprehensive (see with reference to GP data below), for purposes of this study clinical data are considered to be the gold standard against which health survey data may be validated. This is because they are collected on a consistent basis across the jurisdiction of Wales and recorded by professional staff rather than hand-written by survey recipients (as was the case with the WHS). In addition, clinical data are recorded for a comprehensive range of morbidities compared to the limited range which can be captured by a population health survey.

### Objectives

This study aims to exploit the opportunity provided by co-location of anonymised survey- and clinical data to investigate how well self-reported survey data performed compared to the clinical data being made available in the SAIL databank. A secondary objective is to explore whether for some chronic conditions prevalence estimates could more feasibly be made using clinical data. A third objective is to provide a methodological development step towards investigation of many other conditions now recorded in the WHS successor, the NSW. Finally, we hope to contribute to the development of population data science through a complete presentation of the method used to produce these research findings.

This study has been made possible by the SAIL Databank, which provides the ability to link various data sources securely and anonymously in a privacy-protecting platform. Provision to enable informed consent to data linkage was made in the WHS from 2013 onwards so that survey-related research questions can be addressed using anonymised data, which is part of the national e-health records research infrastructure for Wales [3, 4].

## Methods

This is a descriptive cross-sectional study, which applies methodological triangulation to anonymised data from the WHS for 2013 and 2014, with clinical data from 2002 to 2014 in a secure environment. Combining datasets in this way could provide the basis for further analysis of chronic conditions and wellbeing as recorded by the WHS.

### Condition Selection

The incidence of chronic conditions such as stroke and various cancers is well established through national registries [55, 6]. Consequently, the data linkage and triangulation method was applied to a subset of chronic conditions that are present in the survey data and for which registries do not currently exist in Wales. The conditions selected for analysis included ‘angina’, ‘myocardial infarction’ (hereafter referred to as ‘heart attack’, as recorded in the WHS), ‘heart failure’, and ‘asthma’ as recorded via separate tick boxes on the WHS form. Question syntax varied so that respondents were asked if they had ‘ever been treated for’ heart attack, and are ‘currently being treated for’ asthma, angina and heart failure.

### Sample Population – survey data

The WHS samples were selected from the small user version of the Postcode Address File (PAF) provided by Royal Mail. Addresses were randomly sampled and stratified across Wales, with the aim of a minimum sample of 600 adults across each local authority area. These were combined into a single dataset for further analysis. For selected households the WHS also sampled children but consent to linkage was not obtained from them. As a result, no data for children were obtained for this study.

All data were anonymised through a standard split file approach when being acquired into the SAIL Databank. Deterministic matching and validation to a unique identifier (known as an Anonymised Linking Field or ‘ALF’) was applied by the NHS Wales Informatics Service (NWIS) before acquisition into the SAIL Databank.

### Linking to clinical data

Survey records that were not matched to a unique identifier by the anonymization process were removed from the survey dataset at the outset. To maximise the sample size both good and ‘fuzzy’ matches (where it was less clear that records related to the same person) were retained. The two-year WHS analytic sample was then used to select corresponding records from clinical datasets, based on matching unique identifiers contained in each.

The healthcare data linked for this study were the hospital admission data (Patient Episode Database for Wales (PEDW)), welsh population spine of registrations and residence history (Welsh Demographics Service Dataset (WDSD)) and the Welsh Longitudinal General Practice data (WLGP). Records were matched in hospital admission data as far back as 2002 and GP event data back to 2010. Records were also matched with central registry data, as the GP data used covered only around 80% of General Practitioner (GP) surgeries in Wales. In this way any undercount in prevalence from the GP data could be assessed. Specialist physician data were not used as they were not available for this study.

**Figure 1: Data Linkage Using Anonymised Data in SAIL:**
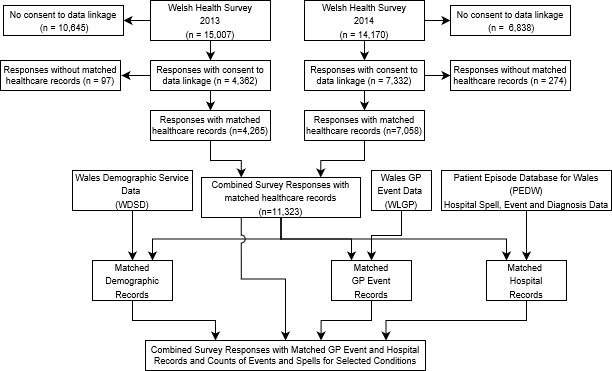


These datasets were linked and combined using SQL DB2 (IBM, Portsmouth, UK) as shown in Figure 1, resulting in a ‘platform file’ containing all relevant data. In this platform file matched records for all survey respondents (n =11,323) were retained for onward analysis of wellbeing for those with and without chronic conditions, including those without any clinical events or diagnoses.

### Identifying chronic conditions

Indication of diagnoses and/or treatments related to ‘angina’, ‘heart attack’, ‘heart failure’ and ‘asthma’ were identified in the clinical data using lists of hospital diagnosis (ICD-10) and GP event (Read) codes (see Supplementary Appendices 1 and 2). For each condition, lists of ICD and Read codes were created based on information from respective online reference sources [7, 8]. For cardiovascular conditions the lists were developed following discussion with a clinician specialising in cardiovascular disease, with particular advice provided on whether GP event codes related exclusively to heart attack or heart failure. For asthma the list of codes was supplemented with an extensive list of medications used to identify cases in the Cognitive development Respiratory Tract Illness and Effects of eXposure (CORTEX) project [9].

All cases of the selected chronic conditions identified were tagged using the lists of ICD and Read codes. For cardiovascular conditions, cases were tagged where clinical codes were solely related to one condition, and not tagged where they could relate to more than one condition - for example instances of oedema which can be related to pregnancy, being overweight, or kidney problems as well as heart failure. Similarly, codes for prescription medications were not used to tag cases of cardiovascular conditions as some medications are used to treat both heart attack and heart failure. In effect an approach was taken to avoid mis-classifying conditions another which prioritised specificity over sensitivity.

All identified cases were tagged in the detailed dataset based on any occurrence of relevant codes as evidence of having been treated for the selected condition. As indicated above, WHS question wording varied so that for heart attack (which asks about ever having been treated), any occurrence of relevant GP event and diagnosis codes were used, whilst for angina, heart failure and asthma (which ask about current treatment), occurrence of relevant GP event and diagnosis codes *in the 12 months prior to interview* were used. Summary counts of relevant events and diagnoses for each survey respondent were extracted using Structured Query Language (SQL) DB2. Further analysis was then carried out in SAS version 9.4 (SAS Institute Inc, Cary, NC) and SPSS 26 (IBM Corp, Armonk, NY) in the SAIL Databank secure environment.

### Data Analysis

A binary flag was derived from responses to relevant survey questions and used to identify cases from the survey data. Survey respondents were identified as cases for the selected chronic conditions based on having had at least one event or diagnosis recorded in the clinical data. Contingency matrices were created, based on clinical data as the ‘gold standard’ against which survey data would be compared. The aim of this approach was to establish groups from these contingency matrices (‘true positive’, ‘false positive’ etc.) for each condition for onward analysis of wellbeing, rather than to define cases per se. Separate case counts were extracted based on whether identified from GP or hospital records, or both.

From the contingency matrices epidemiological measures such as specificity, sensitivity, positive predicted value (PPV) and negative predictive value (NPV) were calculated for each condition based on clinical data as the ‘gold standard’. Indicative prevalence values were calculated with associated confidence intervals (CIs) along with Cohen’s Kappa statistics based on valid responses only (excluding counts for ‘No answer/refused’ responses).

## Results

### Sample Characteristics

With consent for data linkage introduced part-way through 2013 onwards, the numbers of adult with matched health records were 4,362 for 2013 (29.1% of those sampled) and 7,332 for 2014 (51.7% of those sampled). This provided a combined study population of 11,323, for which Table 1 shows the age and gender characteristics. More than half of the sample population was aged over 45, with more females than males for all age groups except 60-74.

**Table 1: Characteristics of WHS Sample with Consent and Successful Data Linkage, 2013 and 2014 Combined:** 

	n (% of Column)					
		
Age Group	Males		Females		Total	
16-29	731	(13.9)	1,014	(16.7)	1,745	(15.4)
30-44	935	(17.8)	1,229	(20.2)	2,164	(19.1)
45-59	1,304	(24.9)	1,559	(25.6)	2,863	(25.3)
60-74	1,565	(29.8)	1,543	(25.4)	3,108	(27.4)
75+	709	(13.5)	734	(12.1)	1,443	(12.7)
All ages	5,244		6,079		11,323
(% of Row)	(46.3)		(53.7)

### Identified Cases

For asthma, more cases were identified from clinical data than were reported by the WHS (see Table 2). For cardiovascular conditions fewer cases were identified from clinical data compared to the WHS, and considerably so (thereby contributing to the variation in PPV values shown in Table 3). Also notable from Table 2 is that for asthma the vast majority of cases were identified from GP events whereas for cardiovascular conditions most were identified via a hospital diagnosis. For ‘currently being treated’ conditions less than 10 percent of cases were identified by both a GP event and hospital diagnosis, whereas over a fifth of ‘ever treated’ heart attack cases were identified in this way. Given that the GP data used covered 80 percent of practitioners, identified case counts could be estimated to be six to eight cases higher for cardiovascular conditions and 269 higher for asthma based on GP records only, though some of these will instead have been picked up through hospital records.

**Table 2: Chronic Condition Cases Identified by GP Event and/or Hospital Diagnosis:** 

Condition	Identified by	Count	% of Total
Angina - Currently being treated	GP Records	32	26
	Hospital Diagnosis	84	68
	Both GP Event and Hospital Diagnosis	8	6
	**Total from Clinical data**	124
	**Total from WHS**	396
Heart Attack – Ever been treated	GP Records	21	18
	Hospital Diagnosis	68	60
	Both GP Event and Hospital Diagnosis	25	22
	**Total from Clinical data**	114
	**Total from WHS**	473
Heart Failure - Currently being treated	GP Records	28	38
	Hospital Diagnosis	39	53
	Both GP Event and Hospital Diagnosis	6	8
	**Total from Clinical data**	73
	**Total from WHS**	191
Asthma - Currently being treated	GP Records	1,079	86
	Hospital Diagnosis	80	6
	Both GP Event and Hospital Diagnosis	97	8
	**Total from Clinical data**	1,256
	**Total from WHS**	1,173

Tables 3-6 show the validation results for the selected chronic conditions.

**Table 3: Respondents ‘Currently’ Treated for Angina at Time of Survey based on WHS and Clinical data:** 

Survey	Clinical data		
		
	Yes	No	Total
Yes	77	319	396
of which:
Female (%)	(42.9)	(43.3)	(43.2)
Male (%)	(57.1)	(56.7)	(56.8)
No	38	10,329	10,367
of which:
Female (%)	(21.1)	(54.0)	(53.9)
Male (%)	(78.9)	(46.0)	(46.1)
No answer/ refused			560
Total			11,323

**Table 4: Respondents Ever Treated for Heart Attack based on WHS and Clinical data:** 

Survey	Clinical data		
		
	Yes	No	Total
Yes	82	391	473
of which:			
Female (%)	(35.4)	(33.2)	(33.6)
Male (%)	(64.6)	(66.8)	(66.4)
No	27	10,359	10,386
of which:
Female (%)	(63.0)	(54.5)	(54.5)
Male (%)	(37.0)	(45.5)	(45.5)
No answer/ refused			464
Total			11,323

**Table 5: Respondents ‘Currently’ Treated for Heart Failure at Time of Survey based on WHS and Clinical data:** 

Survey	Clinical data		
	
	Yes	No	Total
Yes	33	158	191
of which:			
Female (%)	(30.3)	(37.3)	(36.1)
Male (%)	(69.7)	(62.7)	(63.9)
No	33	10,468	10,501
of which:			
Female (%)	(42.4)	(53.8)	(53.8)
Male (%)	(57.6)	(46.2)	(46.2)
No answer/ refused			631
Total			11,323

**Table 6: Respondents ‘Currently’ Treated for Asthma at Time of Survey based on WHS and Clinical data:** 

Survey	Clinical data		
		
	Yes	No	Total
Yes	818	355	1,173
of which:			
Female (%)	(61)	(58)	(60)
Male (%)	(38)	(42)	(40)
No	370	9,220	9,590
of which:			
Female (%)	(53)	(53)	(53)
Male (%)	(47)	(47)	(47)
No answer/ refused			560
Total			11,323

Table 7 summarises the epidemiological measures for each condition. Relative prevalence levels calculated using survey and clinical data are shown, along with Cohen’s Kappa statistics.

Specificity and NPV were high for all conditions, with NPVs in excess of 99 percent for cardiovascular conditions. By contrast, sensitivity varied from 50 percent for heart failure (currently treated) to 75 percent for heart attack (ever treated). The PPV for asthma was notably higher than for cardiovascular conditions.

For cardiovascular conditions there were significant differences in indicative prevalence when based on survey vs. clinical data, ranging from around 10 cases per thousand to over 40 cases per thousand for heart attack. Whether conditions were ‘currently treated’ or ‘ever treated’ made no difference to the disparity in relative prevalence based on the two sources. For cardiovascular conditions indicative prevalence was considerably higher based on survey data compared to clinical data. The divergence in indicative prevalence is reflected in the Kappa statistics which show poor agreement between the two data sources.

For asthma there was no significant difference in indicative prevalence based on the survey vs. clinical data. In addition, the Kappa statistic indicates a fair to good agreement between the data sources.

**Table 7: Respondents ‘Currently’ Treated for Asthma at Time of Survey based on WHS and Clinical data:** 

	Percentages				Prevalence per 1,000 from		Kappa
			
	Sensitivity	Specificity	PPV	NPV	Clinical data	Survey	Statistic
Angina – Currently being treated	66.96	97	19.44	99.63	11	35	0.290
(CIs)					(9/13)	(32/38)
Heart Attack – Ever been treated	75.23	96.36	17.34	99.74	10	42	0.270
(CIs)					(8/12)	(38/45)
Heart Failure – currently being treated	50	98.51	17.28	99.69	6	17	0.250
(CIs)					(5/8)	(15/19)
Asthma – currently being treated	68.86	96.29	69.74	96.14	105	104	0.655
(CIs)					(99/111)	(98/109)

### Missing Data

For the WHS questions on asthma and cardiovascular conditions non-response was between four and five percent overall (see Table 8), and slightly lower for heart attack (‘ever been treated’) than ‘currently being treated’ conditions. The issue of missing data and intentional non-response is discussed further below.

**Table 8: Non-Response to Selected Chronic Condition Questions, Matched WHS Records, 2013 and 2014:** 

WHS question	All Survey Respondents	
	Count	Percentage
Angina – Currently being treated	560	4.95
Heart Attack – Ever been treated	464	4.10
Heart Failure – Currently being treated	631	5.57
Asthma – Currently being treated	560	4.95

## Discussion

Linking health survey, GP and hospital data was relatively straightforward and generated an extensive and expedient dataset for survey validation purposes. Crosstabulation was similarly uncomplicated using the statistical software available in the secure environment provided by the databank.

The validation results for some of the selected conditions are disappointing. In particular the PPV results achieved for cardiovascular conditions (17-19 percent) are low compared to those for asthma, and applying the method for separate cardiovascular conditions was less successful as it was not possible to use medication codes to distinguish between them. As a consequence, there were significant differences in indicative prevalence for cardiovascular conditions based on the two data sources. Although the sets of ICD and Read codes used were developed with expert advice from a clinician, the result was low PPV levels for separate cardiovascular conditions. An alternative approach may have been to validate positive cardiovascular cases from the survey against occurrence of relevant codes in clinical data, then with the results for each condition use machine learning to identify non-self-reporting cases in the remaining data. This would have been more time-consuming but also possibly more effective in identifying those not self-reporting their condition.

The method was more effective for asthma, with a PPV of nearly 70 percent and evidence of better agreement between the data sources, possibly due to better diagnosis and/or more clearly defined interventions for asthma. Most of the difference though is understood to be due to the use of medication codes to identify cases as an initial test run without them yielded a PPV below those for cardiovascular conditions. This indicates that GP read code lists need to include those for medications to identify cases, whether cardiovascular or asthma-related.

These results show that it is possible to use an algorithm to identify chronic disease prevalence from EHRs, but on a qualified basis, e.g. where a list of medication codes are available to identify cases. Further, it is possible to use a data linking method to validate population health survey- with clinical data for chronic conditions, but relative prevalence can vary widely depending on how the conditions are defined. So a data linking method may feasibly be used to estimate prevalence in the absence of a disease register. Where a generally accepted and definitive code list does not exist for a condition, considerable variation in prevalence may be expected. Further, datasets may variously be more suitable for identifying specific chronic conditions e.g. GP rather than hospital records for asthma, as shown in Table 2. This may be due to the way medications for such conditions are coded by GPs. Equally, the method applied did not utilise data from specialist physician or outpatient departments and closer agreement in prevalence may be possible if such data were included. This may particularly be the case for cardiovascular conditions.

By extension, EHRs may not always be a ‘gold standard’ for validating prevalence of chronic conditions depending on whether the available data extends beyond just GP and hospital admission records. Results may also be affected by survey questions, which in this case required respondents and the researcher alike to surmise the meaning of ‘currently treated’ for some conditions and ‘ever treated’ for another. Lower PPV results for all ‘currently treated’ conditions compared to ‘ever treated’ for heart attack from the same survey may be due to additional cognitive bias via the telescoping effect (whereby remote events appear to have occurred more recently), leading to a tendency to over-report over the short term. From this it could be argued that where linkage and validation of survey and clinical data is expected then survey questions on morbidities should perhaps be in terms of whether treated in the last 12 months as recommended by some for hospitalizations [10]. This would enable clearer definition of recall required from respondents and of the lookback period required for data linking purposes.

This study used a single record from either GP or hospital data to identify cases. The aim of this approach was to define subgroups for each condition (true positives, false positives etc.) to enable onward analysis of relative wellbeing. Arguably a more generally acceptable case definition could be applied based on a minimum of two GP visits or a hospitalisation. Previous case definition work has indicated that agreement between data sources varies across chronic conditions and that a common approach does not necessarily exist [11]. The results of this study are consistent with these findings, although perhaps less clear-cut due to the variation in survey questions used to define the conditions. It is possible that any decrease in cases identified using the more stringent definition may be mitigated when validating for those ‘ever treated’. Equally, it could be argued that if access to specific data is limited then case identification could be implemented based solely on GP records for asthma and just hospital data for the most severe cardiovascular conditions.

Improved criterion validity of survey data for cardiovascular conditions could be obtained if validation was applied for heart failure singly and for ischaemic heart disease as a group (to cover angina and ‘heart attack’ as recorded by the WHS). Some studies have shown that medication codes have been used to identify cardiovascular conditions collectively but not separately [12], and treated them as a grouped predictor for medical expenditure [13]. Other studies have found substantial under-recording of chronic conditions [14, 15] and recommended the use of prescription medication data to improve case identification for heart failure [14]. On the other hand, others have found that use of prescription data did not significantly improve the agreement between clinical and survey data when used to define cases for heart disease [11]. A systematic review has indicated that validation of morbidity is more likely to be provided by conventional chart review than administrative data [16].

This study indicates significant differences in prevalence estimates for distinct cardiovascular conditions when based on survey and clinical datasets. By contrast, prevalence estimates for asthma were closely matched. The asthma findings are consistent with those from a survey of 27 asthma-related datasets which indicated UK-level prevalence of 9.6 percent for patient reported clinician-diagnosed-and-treated asthma at 2010-11 [17]. For cardiovascular conditions a similar level of specificity has been found for heart disease as a group, with a higher PPV but lower sensitivity and lower NPV [18]. Low kappa statistic values have also been found when comparing survey and admin for heart failure (‘ever treated’) [19], although not as low as those found in this study (‘currently being treated’).

This study indicates that there were low levels of missing data for the paper-based WHS, and it could be argued that this slightly undermined the WHS as a measurement tool for prevalence of chronic conditions. Given the proximity of the estimates for asthma it could be argued that the issue of non-response would be avoided completely if prevalence was based on clinical rather than health survey data.The data linking method advocated though enables all available WHS records to be linked with analagous clinical data, regardless of non-response to specific questions so that in principle it is possible to establish ‘true’ prevalence for some conditions using clinical data. Alternatively, by using the data linkage and matching method presented above it may be possible to improve the accuracy of prevalence estimates by augmenting survey estimates with clinical data, as suggested by others [21]. On the other hand this may appear to sidestep intentional non response for the sake of improved statistical quality.

A more fundamental question is whether population surveys or clinical data should be used to estimate prevalence of chronic conditions. Since clinical data can now be warehoused and regularly updated it could be argued that surveys are duplicating clinical data collection for some health conditions. With potentially lengthy interviews of over 30 minutes, survey time could easily be freed up to explore qualitative health issues or other policy issues if self-reported health questions were removed. Alternatively, surveys could be shorter overall to avoid bias due to response fatigue. 

### Strengths and limitations

Relatively few studies apply a data linking approach with clinical data and cross-sectional patient-reported information, and such studies tend to be based on data from the USA and elsewhere [15, 21-25]. A strength of this study is that it is fairly unique in applying a data linking approach to UK-based health survey data. This approach has been suggested and recommended by others in the past [26, 27]. Other strengths are that this study is based on national-level survey data and demonstrates a methodological approach to validating population health survey data using data linkage.

This study has some limitations. As obtaining consent to data linkage was introduced part way through the 2013 tranche of the WHS, results are based on a 21-month reporting period (i.e. April 2013 to December 2014) rather than two complete years’ worth of data. The sample may be affected by selection bias as some population groups may more readily have given consent than others based on relative awareness of how their data would be used pre-GDPR (General Data Protection Regulation). Also at the time of the analysis the WLGP dataset comprised data from around 80 percent of GP practices in Wales, and a small number of cardiovascular cases were identified based on just GP data events, so some results (i.e. observed prevalence) may differ slightly if data for all GP practices were utilised. The availability of specialist physician data would also potentially be helpful. The generalisability of the results at a national level may also be limited by the sample size and possible reporting bias, as already highlighted. Population health surveys, now utilise Computer Assisted Personal Interview (CAPI) and Computer Assisted Self Interviewing (CASI) so that the applicability of some findings may be limited.

## Conclusion

This study set out to develop an approach to disease ascertainment for chronic conditions not recorded by clinical registries in Wales. Results indicate that there are inherent limitations in survey and clinical datasets and ultimately it is difficult to determine whether either should be considered as a gold standard for the estimation of prevalence.

A data linking method may feasibly be used to estimate prevalence for some chronic conditions in the absence of a disease register, but data from specialist physician or outpatient departments may be required to obtain reasonable estimates. The results also indicate the method provides better agreement between data sources where prescribed medications can be used to identify conditions. Plausible sets of clinical codes which include medications are required to identify and validate chronic conditions in linked clinical data.

## Acknowledgments

This study makes use of anonymised data held in the Secure Anonymised Information Linkage (SAIL) system, which is part of the national e-health records research infrastructure for Wales. We would like to acknowledge all the data providers who make anonymised data available for research. We would also like to acknowledge the input of Kevin Mohee as a clinical advisor for cardiovascular conditions.

We acknowledge the support from The Farr Institute (MRC Grant No: MR/K006525/1), which funded the secondment that enabled this study. The Farr Institute is supported by a 10-funder consortium: Arthritis Research UK, the British Heart Foundation, Cancer Research UK, the Economic and Social Research Council, the Engineering and Physical Sciences Research Council, the Medical Research Council, the National Institute of Health Research, the National Institute for Social Care and Health Research (Welsh Assembly Government), the Chief Scientist Office (Scottish Government Health Directorates), the Wellcome Trust.

This work was supported by Health Data Research UK, which receives its funding from HDR UK Ltd (NIWA1) funded by the UK Medical Research Council, Engineering and Physical Sciences Research Council, Economic and Social Research Council, Department of Health and Social Care (England), Chief Scientist Office of the Scottish Government Health and Social Care Directorates, Health and Social Care Research and Development Division (Welsh Government), Public Health Agency (Northern Ireland), British Heart Foundation (BHF) and the Wellcome Trust. The work was also supported by an ESRC award establishing the Administrative Data Research Centre Wales (ES/L007444/1).

## Ethics statement

The National Research Ethics Service has previously agreed that research carried out within SAIL does not require ethical review due to the anonymization process applied to the data. Standard Ethical Approval was nevertheless obtained from Swansea University Medical School Research Ethics Sub-Committee for the purposes of this project, approval number 2017-0020.

## Supplementary Appendices

1. Read- and ICD Codes used to identify cardiovascular patients.
2. Read- and ICD codes used to identify asthma patients.

## Abbreviations

ALF	Anonymised Linking Field
CAPI	Computer Assisted Personal Interview
CASI	Computer Assisted Self Interviewing
CIs	Confidence Intervals
EHR	Electronic Health Record
GP	General Practitioner
ICD-10	International Classification of Diseases Tenth Revision
NHS	National Health Service
NPV	Negative Predicted Value
NSW	National Survey for Wales
NWIS	NHS Wales Informatics Service
PAF	Postcode Address File
PEDW	Patient Episode Database for Wales
PPV	Positive Predicted Value
SAIL	Secure Anonymised Information Linkage
SQL	Structured Query Language
WLGP	Welsh Longitudinal General Practice dataset
WDSD	Welsh Demographics Service Dataset
WHS	Welsh Health Survey
